# A gene network-driven approach to infer novel pathogenicity-associated genes: application to *Pseudomonas aeruginosa* PAO1

**DOI:** 10.1128/msystems.00473-23

**Published:** 2023-11-03

**Authors:** Ronika De, Marvin Whiteley, Rajeev K. Azad

**Affiliations:** 1 Department of Biological Sciences, University of North Texas, Denton, Texas, USA; 2 BioDiscovery Institute, University of North Texas, Denton, Texas, USA; 3 Center for Microbial Dynamics and Infection, School of Biological Sciences, Georgia Institute of Technology, Atlanta, Georgia, USA; 4 Emory-Children’s Cystic Fibrosis Center, Atlanta, Georgia, USA; 5 Department of Mathematics, University of North Texas, Denton, Texas, USA; Drexel University, Philadelphia, Pennsylvania, USA

**Keywords:** *Pseudomonas aeruginosa*, gene co-expression network, pathogenicity, antibiotic resistance

## Abstract

Hard-wired within the genome of a pathogen is the information regarding factors responsible for its pathogenicity. Advances in functional genomics, particularly gene expression analysis, have made possible genome-wide interrogation of a pathogen to decipher pathogenicity-associated genes. Standard protocols assess differential expression of genes during pathogenesis; however, often a conservative approach is taken, which requires expression fold change above an arbitrarily defined threshold and a statistical significance test to infer a gene as differentially expressing. This renders a high-confidence set of differentially expressed genes in pathogenesis, however, at the cost of numerous false negatives. To circumvent this problem and comprehensively catalog pathogenicity-associated genes, we have developed a novel pipeline that uses standard protocol in combination with gene co-expression network of a pathogen constructed using publicly available RNA-Seq data sets. We assessed the efficacy of this pipeline on *Pseudomonas aeruginosa* PAO1, a model bacterial pathogen, highlighting the power of our network-based approach in discovering novel genes or pathways associated with the pathogenesis, or antibiotic resistance of this strain. Complementing standard protocol with a gene network-based method thus elevated the ability to identify pathogenicity-associated genes in *P*. *aeruginosa* PAO1.

**IMPORTANCE:**

We present here a new systems-level approach to decipher genetic factors and biological pathways associated with virulence and/or antibiotic treatment of bacterial pathogens. The power of this approach was demonstrated by application to a well-studied pathogen *Pseudomonas aeruginosa* PAO1. Our gene co-expression network-based approach unraveled known and unknown genes and their networks associated with pathogenicity in *P. aeruginosa* PAO1. The systems-level investigation of *P. aeruginosa* PAO1 helped identify putative pathogenicity and resistance-associated genetic factors that could not otherwise be detected by conventional approaches of differential gene expression analysis. The network-based analysis uncovered modules that harbor genes not previously reported by several original studies on *P. aeruginosa* virulence and resistance. These could potentially act as molecular determinants of *P. aeruginosa* PAO1 pathogenicity and responses to antibiotics.

## INTRODUCTION

Differential gene expression analysis has been extensively used as a standard procedure to investigate key genetic players responsible for specific phenotypes elicited by bacteria, including survival, growth, drug resistance, and pathogenicity under different conditions. Conventional approaches for the identification of differentially expressing genes (DEGs) under various conditions depend on expression fold change and statistical tests. Fold change signifies the factor by which the expression level of a gene changes between two different conditions and often provides biologically meaningful insights for such altered expression. Fold change does not take variability into account and does not guarantee reproducibility of the results for differential gene expression analysis (1). It was reported that variability of fold change of a gene is inversely proportional to the expression level of that gene (1, 2). This may result in lowly expressed genes displaying higher fold change, whereas the inverse is true for highly expressed genes, which can confound the analysis of DEGs. Therefore, statistical significance test is often used along with fold change to catalog DEGs with higher confidence (3–6). While the inclusion of a statistical significance test augments the ability to identify DEGs with greater confidence, it may still cause a generation of undesirably numerous false negatives. To circumvent this problem and robustly detect DEGs, we have developed a novel pipeline that combines the traditional approach with a gene co-expression network-based approach to catalog genes differentially expressing under a specific condition. A gene co-expression network describes functional relationships among genes, in terms of correlation in their expression patterns across different conditions, assessed in a pairwise manner (7). The nodes in a gene co-expression network represent genes and the edges connecting nodes represent association, assessed in terms of correlation in the expression patterns of genes represented by the nodes (8, 9).

Co-expression networks have been used to characterize genes with yet unknown functions, bacterial pathogenesis, antimicrobial resistance, and biological pathways as well as their interactions in different bacterial models including *Escherichia coli*, *P. aeruginosa*, *Staphylococcus aureus*, *Vibrio cholerae*, and *Mycobacterium tuberculosis* (10–18). These studies have found that functional annotations (i.e., biological processes and pathways) represented by modules identified from co-expression networks are useful for understanding the functions of genes encoding hypothetical (yet uncharacterized) proteins. Previous studies have also reported the enrichment of gene ontology (GO) terms signifying certain biological processes in the modules (19–22), and further, the correlations in the expression patterns of genes within modules were found to be congruent with pathway annotations of the genes (19, 21, 22). Some studies (10, 11, 23–34) have interrogated the co-expression networks to decipher the functions of unannotated genes and the pathways they represent using the guilt-by-association approach (35–37). Therefore, we posit that such networks can be leveraged to identify novel genes that differentially express under certain conditions but are prone to be missed by standard protocols based on conservative statistical tests. This is based on the premise that if such genes, displaying twofold or more expression change yet deemed not significant, associate with genes in modules enriched in differentially expressing genes, this could be indicative of their differential expression deciphered through our systems (network)-level approach, as further discussed below.

**Fig 1:**
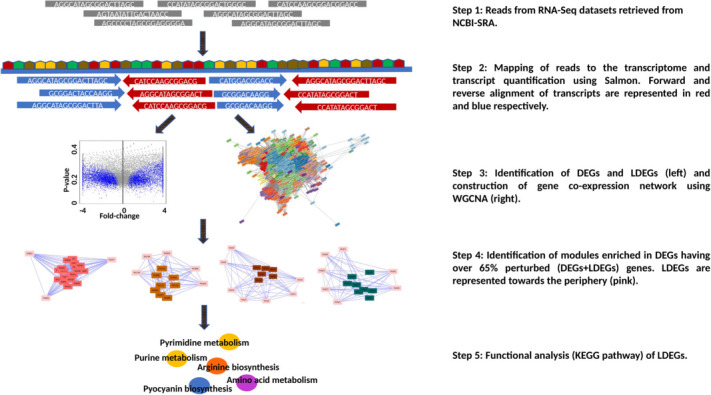
Schematic diagram of the gene co-expression network-based pipeline for the identification and functional characterization of likely differentially expressing genes (LDEGs) that are missed by standard protocols for differential gene expression analysis. This pipeline involves retrieving RNA-Seq data sets from NCBI-SRA (Step 1) and alignment of reads onto the PAO1 transcriptome using the tool Salmon (Step 2). Next, a PAO1 gene co-expression network is constructed, and differential gene expression analysis is performed to identify DEGs (Step 3). LDEGs were identified in modules enriched in DEGs and having over 65% perturbed genes (Step 4). Finally, a functional analysis of LDEGs is performed (Step 5). DEGs refer to differentially expressing genes.

We demonstrate here the efficacy of the proposed pipeline by applying it to expression data sets obtained for a model pathogen, *Pseudomonas aeruginosa* PAO1 (*P*. *aeruginosa* PAO1 or simply PAO1 henceforth). Our pipeline (Fig. 1) uses RNA-Seq data sets of an organism (strain or species) representing several conditions to construct a gene co-expression network and then partition the network into gene modules, with each module containing genes that are tightly linked by co-expression. Given an expression data set for the organism representing a specific condition, as well as the corresponding control data set, DEGs identified using both fold change and statistical test are mapped onto the gene co-expression network. This allows the identification of gene modules in the network, which are significantly enriched in the DEGs (*P*-value < 0.05). For each enriched module, we focused on the non-DEGs with fold change of two or greater. For our analysis, we considered these genes to be also perturbed in expression, in addition to the DEGs. DEG-enriched modules comprise about two-thirds or more (over 65%) genes exhibiting perturbed expression (i.e., the DEGs and non-DEGs with twofold or more expression change) were interrogated to determine functions that were enriched in these modules, as well as infer potential functions of the non-DEGs displaying perturbed expression in these modules using information gleaned from Kyoto Encyclopedia of Genes and Genomes (KEGG) pathway database and literature studies.

In application to the model pathogen, *P*. *aeruginosa* PAO1, we show the usefulness of our integrative approach in deciphering novel genetic factors and biological pathways associated with pathogenicity and antibiotic response in a pathogen, which is otherwise missed by conventional approaches of differential gene expression analysis. *P*. *aeruginosa* was used in this study as it is a well-known bacterial pathogen that is widespread in natural environments and is the causative agent of a broad spectrum of diseases in animals and humans, such as urinary infection, respiratory infection, and septicemia (38, 39). Furthermore, its ability to survive under tremendous exposure to different antibiotics renders it very difficult to treat in clinical settings. Understanding of genes and pathways regulating PAO1 pathogenicity as well as its response to antibiotics is central to deconstructing the mechanisms that drive its pathogenesis and resistance. A systems-level approach is clearly needed to investigate the networks of genes regulated during pathogenesis and upon exposure to antibiotics in bacterial pathogens, such as *P*. *aeruginosa*. This study is a step forward in this direction and holds great promise in unraveling bacterial pathogens through a systems-level investigation.

## MATERIALS AND METHODS

### Transcriptomic data processing and analysis

We obtained the expression profile data sets for *P. aeruginosa* PAO1 from the National Center for Biotechnology Information (NCBI) GEO database (https://www.ncbi.nlm.nih.gov/geo/) (40). The RNA-Seq data sets for the PAO1 strain representing many different conditions were retrieved from the NCBI SRA database (https://www.ncbi.nlm.nih.gov/sra) and converted to fastq files using the SRA Toolkit (41). A total of 834 samples available at NCBI SRA were downloaded. Transcriptome indexing was performed using Salmon v1.4.0 (42). For each sample, the reads were mapped onto the reference *P. aeruginosa* PAO1 transcriptome and then the transcript abundance was quantified and normalized using Salmon v1.4.0 (43). We used Trimmomatic v0.39 (44) to trim adaptor sequences and low-quality regions (minimum length of 35 nt). After adapter removal and trimming, we evaluated the quality of the reads using FastQC v0.11.8 (45). For gene co-expression network construction, we performed preprocessing of transcriptomic data to remove low-quality samples with less than 50% of reads mapping onto the reference transcriptome, leaving 261 samples used for further analysis (accession numbers of these samples are provided in Web Material File 1). The accession numbers of *P. aeruginosa* RNA-Seq data sets that were used for discovering novel genes differentially expressing during wound infections, cystic fibrosis, and response to azithromycin are SRP033652, SRP159291, and SRP264926, respectively.

### Gene co-expression network construction

Expression profile data sets of *P. aeruginosa* PAO1 were used to construct a gene co-expression network using the weighted gene correlation network analysis (WGCNA) (8) package. The gene co-expression network was constructed using the blockwiseModules function to generate a signed network under a soft-thresholding power of 5, minimum module size of 10, merge cut height of 0.1, and deep split of 2. We examined the characteristics of this network, particularly, the scale-free topological properties that are intrinsic to a plethora of real-world networks (46, 47), including biological networks as reported by various studies (48–51). Other topological properties examined here include network density, mean connectivity, and mean clustering coefficient. Following the network construction, gene modules were retrieved using WGCNA. A gene module comprises genes that display high correlation in their expression patterns and are, therefore, associated by their co-expression within the network (19–22, 52–55). These modules were examined for the enrichment of operon-harbored genes as genes in an operon are co-regulated, that is, they are functionally coupled and so should be recovered together within a co-expression module of the network. Enrichment of operon genes in modules was assessed based on a statistical significance test for fold enrichment (significance level set at 0.05). “Hub” genes, that is, those with a high degree of connectivity and therefore deemed functionally important (56), were identified based on weighted betweenness and closeness scores for the genes using the R package Centiserve v1.0.0 (57). The betweenness score is a measure of the number of shortest paths between every two other genes that pass through a certain gene (58). A higher betweenness score for a gene indicates higher connectivity of the gene. Closeness score is a measure of the degree to which a gene is near to all of the other genes in a network (58); genes with a higher closeness score indicate that they are well-connected in the network (59). Such genes with high betweenness and closeness scores could, therefore, be more biologically informative and prioritized for further downstream analyses (60). Cytoscape (61) was used for the visualization of the co-expression network containing edges with weight ≥0.04.

### Identification of DEGs during *P. aeruginosa* pathogenesis and antibiotic treatment

We performed differential gene expression analysis to understand *P. aeruginosa* pathogenesis in cystic fibrosis lungs using DESeq2 (62). Genes that displayed expression fold change of two or more and false discovery rate (FDR) adjusted *P*-value ≤ 0.05 were deemed differentially expressing during infection in cystic fibrosis lungs. We used DEGs that were identified in previous studies for murine wound infections (acute and chronic) (63) and azithromycin treatment (64). DEGs were identified using DESeq (65) and DESeq2 (62) in the murine wound infections (acute and chronic) (63) and azithromycin treatment (64) studies, respectively.

### Mapping of DEGs onto the modules

DEGs were identified using the criteria of expression fold change and statistical significance test. These DEGs were then mapped onto the gene modules generated using WGCNA. This analysis provided information about the DEG containment of each module in the network for each considered study. The DEG containment of some modules ranges from none to a few DEGs, while other modules consist of a large number of DEGs.

### Identification of modules enriched in DEGs

Modules enriched in differentially expressing genes were determined by assessing the statistical significance of fold enrichment of DEGs in modules with fold enrichment > 1. This was accomplished by performing the Fisher’s test and the modules with *P*-value ≤ 0.05 were deemed significantly enriched. We did not use a higher threshold for fold enrichment to avoid losing any modules that could be potentially important for bacterial pathogenicity or survival under certain conditions.

### Identification of additional genes that are likely differentially expressing but are missed by statistical tests

We considered DEG-enriched modules and because of the DEG enrichment, we deemed these modules that could likely be representing pathways or subnetworks of pathways to be differentially expressing. However, these modules also harbor genes that were deemed non-DEG by statistical tests used in the respective studies. Because they reside within the DEG-enriched modules, it is plausible that many of these genes may be participating in the same biological processes as the DEGs, and these could be on the same pathways as the DEGs. Based on this premise, we posit that among these genes, those with expression fold change of two or more are LDEGs that were missed by the standard statistical approaches used in the original studies. We further selected those DEG-enriched modules that have a large majority (over 65%) of the genes that are DEGs or LDEGs. We investigated LDEGs in the DEG-enriched modules for their potential roles in aiding bacterial pathogenicity or survival under certain conditions.

### Functional enrichment analysis of modules containing LDEGs

To understand the roles of the modules enriched in DEGs and containing a large majority (>65%) of genes either differentially expressing or likely differentially expressing during wound (acute and chronic) infections, infections in cystic fibrosis lungs, and azithromycin treatment, we performed functional (gene ontology [GO] term and KEGG pathway) enrichment analysis of these modules using DAVID (66). Those with GO terms and/or KEGG pathways displaying fold enrichment > 1 and FDR adjusted *P*-value < 0.05 were identified as significantly enriched.

### Use of Tn-Seq and machine learning to prioritize LDEGs identified using the network-based pipeline

To prioritize LDEGs for further research toward the goal of identifying novel genes involved in pathogenesis and antibiotic resistance, we cataloged LDEGs that were further supported by Tn-Seq, machine learning, or both (63, 67).

### Pathway annotation for LDEGs

LDEGs were annotated for pathways they are part of using KEGG pathway database (68) as well as based on literature studies. This analysis sheds light on the functions of LDEGs and helps us decipher if LDEGs are parts of some known pathways or play roles in yet unknown pathways.

## RESULTS AND DISCUSSION

### Retrieval of *P. aeruginosa* PAO1 DEGs from acute burn and chronic surgical wound infection studies

To understand transcriptional programming in *P. aeruginosa* PAO1 during pathogenesis from a network perspective, we first retrieved PAO1 transcriptomic data sets from a study on acute wound infection and chronic wound infection (NCBI SRA accession: SRP033652) (69). We obtained a list of 829 PAO1 DEGs from an acute burn surgical wound infection and 1,100 PAO1 DEGS from a chronic surgical wound infection (Web Material File 1).

### Identification of *P. aeruginosa* DEGs during infection of cystic fibrosis lungs

We used RNA-Seq data set from a study on *P. aeruginosa* infection of cystic fibrosis lungs (NCBI SRA accession: SRP159291) (70). We performed differential gene expression analysis and identified 2044 DEGs during the infection (Web Material File 1).

### Retrieval of *P. aeruginosa* PAO1 DEGs from an azithromycin exposure study

We retrieved RNA-Seq data set from a study on the exposure of PAO1 strain to azithromycin (NCBI SRA accession: SRP264926) (71). We then obtained a list of 3,054 DEGs (Web Material File 1) from this study on the exposure of PAO1 to azithromycin (71). Differential expression was determined by comparing azithromycin-exposed PAO1 with PAO1 grown in a serum medium.

### Gene co-expression network construction

**Fig 2:**
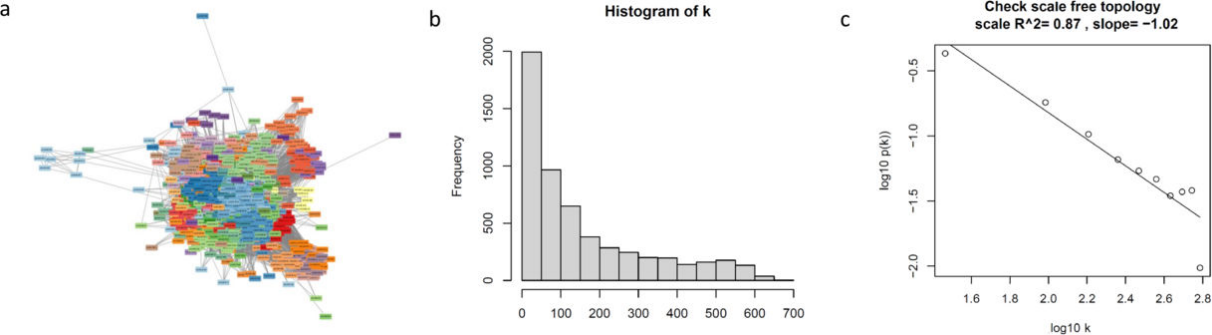
(a) *Pseudomonas aeruginosa* PAO1 gene co-expression network. The network comprises 48 gene modules, which are shown in different colors. The network was constructed using WGCNA, and the gene modules were retrieved at a soft-thresholding power of 5, minimum module size of 10, merge cut height of 0.1, and deep split of 2 (b) The degree distribution of the PAO1 co-expression network is shown as a histogram of degree (number of connections for a node, *k*), which reveals that many genes have few connections and few genes have many connections in the network. (c) The log-log plot depicting the characteristic of network connectivity, highlighting the approximate linear relationship of *P*(*k*) with *k* on the log-log scale and a high *R*^2^ value of 0.87, which indicates an approximate scale-free topology of the network. *P*(*k*) refers to the fraction of nodes in the network having *k c*onnections to other nodes.

A gene co-expression network for *P. aeruginosa* PAO1 was constructed using RNA-Seq data sets for PAO1 grown in diverse conditions by employing the network construction tool WGCNA (Fig. 2). The parameters used for the construction of this network are provided in Materials and Methods. Biological networks, such as gene regulatory, metabolic, and protein-protein interaction networks, often display scale-free topology, with the degree distribution approximating a power-law distribution (72). We examined the *P. aeruginosa* PAO1 co-expression network for scale-free topological features. In such networks, the probability *P*(*k*) of degree *k* (i.e., *k* connections to a node) follows a power-law relationship, $P\left(k\right)\sim k^{-\gamma }$ , where the parameter γ ≥1. These networks self-organize into a scale-free topology, wherein the networks have a large number of nodes with low connectivity and a few nodes with high connectivity. The degree distribution of the *P. aeruginosa* PAO1 co-expression network is shown in Fig. 2b as a histogram of *k* and in Fig. 2c as a log-log plot depicting the power-law relationship $P\left(k\right)\sim k^{-\gamma }$. These data conform well with the power-law behavior, with the histogram (Fig. 2b) showing many genes with few connections and few genes with many connections in the network. Furthermore, the approximate linear relationship of *P*(*k*) with *k* on the log-log scale and a high *R*^2^ value of 0.87 (Fig. 2 c) indicate an approximate scale-free topology of the co-expression network. The other topological properties analyzed here include network density, mean connectivity, and mean clustering coefficient, which have previously been examined for biological networks (50, 51, 73, 74). Connectivity is determined by WGCNA using the adjacency profile of a gene in the adjacency matrix, i.e., the summation over connection strengths to other nodes. Mean connectivity is the average of the adjacencies (or connection strengths) of all genes with the other genes in the network. The network density is obtained as the ratio of the total connectivity (number of edges) between genes (nodes) in a network and the number of all possible connections among the genes in the network. The clustering coefficient signifies the degree to which neighbors of the nodes are interconnected to each other in the network. The *P. aeruginosa* PAO1 co-expression network has a density of 0.027, a mean connectivity of 154.75, and a clustering coefficient of 0.138.

This network was used to identify gene modules and pathways in PAO1, which play important roles during pathogenesis and upon the exposure of PAO1 to an antibiotic (azithromycin). A total of 48 co-expression modules were identified in this network (Fig. 2a; Table 1). To evaluate the validity of the modules identified in this network, we identified operons in PAO1 using the tool MicrobesOnline (75) (Web Material File 2), and performed enrichment analysis for operon genes in the co-expression modules. As expected, the enrichment of genes for over two-thirds (67.57%) of operons with three or more genes was observed in the modules of the PAO1 network. This increased to 73.14%, 78.44%, and 82.29% of operons with at least four, five, and six genes respectively (Web Material File 3). We further identified the top 50 hub genes in the network (Web Material File 4), based on their weighted betweenness and closeness scores. GO and pathway enrichment analyses of these hub genes revealed their roles in protein secretion (type III), sulfur metabolism, and biofilm formation (Web Material File 5).

**TABLE 1:** Enriched GO terms and KEGG pathways in modules 1–48

Module	Biological process	Molecular function	Cellular component	KEGG pathway	KEGG fold enrichment
1	Sphingosine catabolic process	Fimbrial usher porin activity	Acetolactate synthase complex	Biosynthesis of various other secondary metabolites	4.87
2	L-arginine import across plasma membrane	1,4-dihydroxy-2-naphthoyl-CoA thioesterase activity	Cell division site	Vancomycin resistance	2.95
3	Lipopolysaccharide export	Translation release factor activity, codon specific	Chromosome	Citrate cycle (TCA cycle)	4.38
4	Branched-chain amino acid catabolic process	Hydrolase activity, hydrolyzing O-glycosyl compounds	Small ribosomal subunit	Starch and sucrose metabolism	7.75
5	L-glutamate import	Alkaline phosphatase activity	Type IV pilus	Bacterial chemotaxis	4.07
6	Metal ion transport	Cytidine triphosphate (CTP) synthase activity	Type II protein secretion system complex	Nucleotide excision repair	11.80
7	Alginic acid acetylation	Alginate synthase activity	Methylcrotonoyl-CoA carboxylase complex	Fructose and mannose metabolism	12.82
8	Cytoplasmic translation	Large ribosomal subunit rRNA binding	Cytosolic small ribosomal subunit	Ribosome	17.79
9	Protein maturation by iron-sulfur cluster transfer	Cytochrome-c oxidase activity	Respiratory chain	Porphyrin metabolism	8.47
10	Regulation of L-arginine import	RNA polymerase binding	Cytosolic large ribosomal subunit	Protein export	12.71
11	dUMP biosynthetic process	–	Plasma membrane protein complex	Glycerolipid metabolism	15.87
12	Alkanesulfonate catabolic process	Taurine dioxygenase activity	Membrane	Sulfur metabolism	25.68
13	DNA-templated transcription, initiation	Peroxiredoxin activity	–	–	–
14	Ethanolamine catabolic process	Ethanolamine ammonia-lyase activity	Ethanolamine ammonia-lyase complex	Pentose phosphate pathway	12.32
15	Alkanesulfonate transport	Structural constituent of ribosome	Cytoplasm	Ribosome	8.33
16	Leucine import across plasma membrane	Toxin-antitoxin pair type II binding	Integral component of membrane	Biofilm formation—*Pseudomonas aeruginosa*	8.36
17	Terpene catabolic process	Citronellyl-CoA dehydrogenase activity	Integral component of membrane	Geraniol degradation	46.68
18	Polysaccharide biosynthetic process	NADH dehydrogenase activity	Periplasmic side of plasma membrane	O-antigen nucleotide sugar biosynthesis	37.03
19	Acetyl-CoA biosynthetic process	ATP binding	ATP-binding cassette (ABC) transporter complex	Taurine and hypotaurine metabolism	21.56
20	Gamma-aminobutyric acid catabolic process	Glycine dehydrogenase (cyanide-forming) activity	Cytoplasm	Cyanoamino acid metabolism	20.81
21	Spermidine catabolic process	Structural molecule activity	Bacterial-type flagellum hook	Flagellar assembly	20.21
22	Cellular response to iron ion starvation	Phosphopantetheine binding	Cytosol	Pentose phosphate pathway	28.34
23	Pyoverdine biosynthetic process	Heme transporter activity	Efflux pump complex	Biosynthesis of amino acids	6.79
24	Glycerol metabolic process	Potassium-transporting ATPase activity	–	Two-component system	3.37
25	Organic phosphonate metabolic process	Monooxygenase activity	–	Phosphonate and phosphinate metabolism	102.87
26	Cellular response to calcium ion	Chaperone binding	Type III protein secretion system complex	Bacterial secretion system	16.41
27	Beta-alanine biosynthetic process	–	Extracellular region	–	–
28	Bacteriocin biosynthetic process	–	–	–	–
29	Histidine catabolic process	Histidine ammonia-lyase activity	–	Histidine metabolism	45.92
30	Secretion	Oxidoreductase activity	Cytosol	Two-component system	6.03
31	Siderophore transmembrane transport	Siderophore uptake transmembrane transporter activity	Intrinsic component of cell outer membrane	D-amino acid metabolism	24.64
32	Branched-chain amino acid biosynthetic process	Cytochrome o ubiquinol oxidase activity	Cytochrome o ubiquinol oxidase complex	Valine, leucine, and isoleucine biosynthesis	52.21
33	Protein transport by the Sec complex	Xenobiotic transporter activity	Extracellular space	Quorum sensing	12.4
34	Cytolysis	–	–	–	–
35	4-amino-4-deoxy-alpha-L-arabinopyranosyl undecaprenyl phosphate biosynthetic process	–	–	Cationic antimicrobial peptide (CAMP) resistance	39.68
36	Nitrate metabolic process	Nitrate reductase activity	Nitrate reductase complex	Nitrogen metabolism	22.44
37	–	–	–	–	–
38	–	Oxidoreductase activity	–	–	–
39	Protein secretion by the type VI secretion system	–	–	Biofilm formation—*Pseudomonas aeruginosa*	13.65
40	DNA integration	Transposase activity	Protein-DNA complex	–	–
41	Protein secretion by the type III secretion system	–	–	Bacterial secretion system	21.10
42	Pyochelin biosynthetic process	Phosphopantetheine binding	–	Biosynthesis of siderophore group nonribosomal peptides	212.57
43	Secondary metabolite biosynthetic process	Anthranilate synthase activity	–	Phenazine biosynthesis	57.86
44	–	Copper ion binding	–	–	–
45	Autolysis	–	–	–	–
46	–	–	–		
47	–	Dioxygenase activity	Extracellular region	Aminobenzoate degradation	43.76
48	Protein refolding	Unfolded protein binding	HslUV protease complex	RNA degradation	110.22

### Module annotation and identification of LDEGs using the PAO1 network

A module consists of genes that have similar expression patterns under diverse conditions, and it is therefore important to characterize the biological processes or pathways represented by the modules. To this end, we performed GO and KEGG pathway enrichment analyses to identify significantly enriched functional terms in the 48 modules of the network using DAVID (https://david.ncifcrf.gov/) (76). GO terms providing information about cellular localization, molecular function, and biological process were obtained. We annotated each of the 48 modules based on fold enrichment of GO terms and pathways (Web Material Files 6 and 7).

### PAO1 acute and chronic wound infections

**Fig 3:**
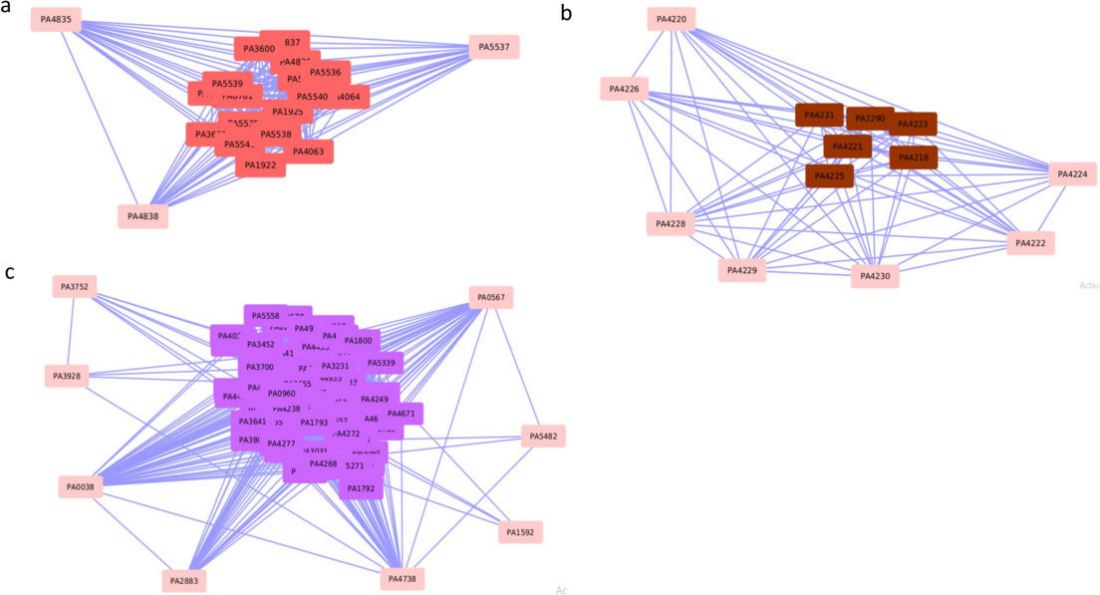
LDEGs from a chronic wound infection RNA-Seq data set, which were found following mapping of genes with expression fold change > X onto the *P. aeruginosa* PAO1 gene co-expression network and identification of DEG-enriched modules that have over 65% genes with expression fold change > X (see text). The LDEGs are shown here in DEG-enriched module (a) #31, (b) #42, and (c) #8. LDEGs in each module are highlighted in pink.

The DEGs identified for acute burn wound infection were mapped onto the PAO1 co-expression network to identify modules that contain these genes. We focused particularly on those modules that have an enrichment of DEGs and consist of a large majority of genes that are either DEGs or LDEGs (over 65%) (Web Material Files 8 and 9). Note that for calling LDEGs in the DEG-enriched modules, we used the expression fold change criterion used in the original study (69), that is, fourfold or more expression change. As a co-expression module could be representing a biological pathway or a network of pathways, we, therefore, focused on these DEG-enriched modules with a large majority of perturbed genes (over 65%). We identified such a module, namely, module 42, in both acute and chronic wound infections, and modules 8 and 31 in only chronic wound infection (Fig. 3; Web Material File 9). To understand the role of these modules in acute and chronic wound infections, we performed functional (GO term and KEGG pathway) enrichment analysis of these modules (Web Material File 10). In module 8, the cytoplasmic translational processes and ribosome were the significantly enriched GO terms and pathways, respectively, during the chronic wound infection; expression analysis revealed that the respective genes were downregulated. Downregulation of translation/ribosomal transcripts suggests reduced bacterial growth rate during the chronic wound infection. In module 31, we found enrichment of siderophore transmembrane transport (GO process) and D-amino acid metabolism (KEGG pathway), which were upregulated during the chronic wound infection. In module 42, pyochelin (siderophore) biosynthesis and biosynthesis of siderophore group nonribosomal peptides were found to be the enriched GO and pathway terms, respectively, and the corresponding genes were found upregulated during acute and chronic wound infection. Siderophores including pyochelin chelate iron from mammalian hemoproteins and, therefore, they are required for bacterial survival in mammalian hosts (77–81). Pyochelin has been associated with chronic infections (82) and may be contributing to bacterial fitness in mammalian hosts. As modules 31 and 42 are enriched in siderophore biosynthesis and transport, it is likely that these modules play important roles in the PAO1 infections.

**Fig 4:**
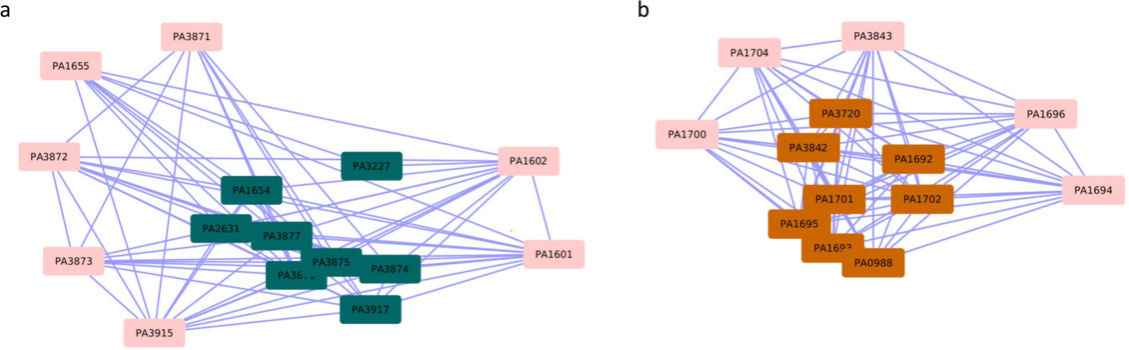
(a) Module 36 and (b) module 41 are not significantly enriched in DEGs but comprise over 65% of genes that are either DEGs or LDEGs from a chronic wound infection PAO1 expression data set. LDEGs in each module are highlighted in pink.

Interestingly, we found two modules, 36 and 41 (Fig. 4a and b), which are not significantly enriched in DEGs for the chronic wound infection but comprise over 65% genes that are either DEGs (26.66% and 30.76%, respectively) or LDEGs (46.66% and 38.46%, respectively) (Table 2; Web Material File 9). As these modules are not enriched in DEGs but have about two-thirds of the genes that are either DEGs or LDEGs, they could potentially be associated with pathogenicity, which needs to be investigated further to establish their roles in PAO1 pathogenesis. To get insights into the potential roles of these non-enriched modules in the chronic wound infection, we performed GO and KEGG pathway enrichment analysis of these modules (Web Material File 10). In module 36, we found GO enrichment of processes such as nitrate metabolism and anaerobic respiration that were found to be upregulated. Pathway enrichment analysis revealed enrichment of nitrogen metabolism, which was found to be upregulated. As anaerobic respiration and energy metabolism were recently associated with chronic wound infection (83), we posit that module 36 is critical to chronic wound infection. We found enrichment of GO terms bacterial secretion (type III) and pathogenesis in module 41, with the respective genes found to be upregulated. Enrichment of bacterial secretion system (upregulated) in module 41 was also indicated by pathway enrichment analysis. Type III secretion system is used to inject effector proteins into host cells and has been associated with chronic wound infection (69, 83). This supports the plausible involvement of module 41, enriched in type III secretion system and pathogenesis terms, in the chronic PAO1 infection.

**TABLE 2:** DEG-enriched modules with over 65% of genes either DEGs or LDEGs in acute burn wound, chronic surgical wound, and cystic fibrosis (CF) lungs infection

Module	DEG count	LDEG count	Genes (DEGs + LDEGs) (%)	Fold enrichment	Fisher’s test (*P*-value)	Infection type	Enriched/non-enriched
42	12	0	0.923	6.169	2.021e-10	Acute	Enriched
8	49	8	0.750	3.227	3.510e-21	Chronic	Enriched
31	16	3	0.950	4.004	5.940e-10	Chronic	Enriched
42	5	7	0.923	1.925	0.047	Chronic	Enriched
9	48	5	0.697	1.693	2.631e-11	CF lungs	Enriched
14	42	7	0.731	1.680	6.550e-10	CF lungs	Enriched
16	53	0	0.803	2.153	1.172e-19	CF lungs	Enriched
22	29	0	0.963	2.046	2.468e-10	CF lungs	Enriched
23	22	3	0.862	2.034	4.719e-08	CF lungs	Enriched
26	20	0	0.800	2.145	4.109e-08	CF lungs	Enriched
28	13	3	0.727	1.584	0.00242	CF lungs	Enriched
30	20	0	0.909	2.437	4.277e-10	CF lungs	Enriched
31	20	0	1	2.681	3.318e-12	CF lungs	Enriched
36	9	2	0.733	1.608	0.00680	CF lungs	Enriched
41	10	2	0.923	2.062	2.312e-04	CF lungs	Enriched
42	13	0	1	2.681	3.514e-08	CF lungs	Enriched
36	4	7	0.733	1.334	0.289	Chronic	Non-enriched
41	4	5	0.692	1.540	0.247	Chronic	Non-enriched
44	6	2	0.666	1.340	0.0968	CF lungs	Non-enriched

### *P. aeruginosa* infection in cystic fibrosis lungs

We mapped DEGs identified during the infection of *P. aeruginosa* in cystic fibrosis lungs onto the PAO1 network and identified 12 modules (#9, 14, 16, 22, 23, 26, 28, 30, 31, 36, 41, and 42) that were enriched in DEGs and contained over 65% DEGs or LDEGs (Table 2; Web Material File 9). To investigate the roles of these modules during the infection in cystic fibrosis lungs, we performed GO and pathway enrichment analyses (Web Material File 10). Module 9 was identified to be enriched in upregulated GO processes such as denitrification and protein maturation by iron-sulfur cluster transfer and in downregulated pathways including porphyrin metabolism. We found enrichment of upregulated processes and pathways including carbohydrate (pentose phosphate pathway) and ethanolamine metabolism in module 14. Upregulation of denitrification was previously associated with microaerobic or anaerobic environment of cystic fibrosis lungs (84, 85). Downregulation of enriched processes and pathways involved in bacterial secretion of amino acids was observed in module 16. Module 22 was enriched in upregulated GO processes including pyoverdine biosynthetic process and cellular response to iron starvation. Upregulation of pentose phosphate pathway was also detected in this module. Pyoverdine biosynthetic process was also found to be upregulated and enriched in module 23. This module was also enriched in amino acid biosynthetic pathway that was upregulated. Pyoverdine chelates iron from mammalian hemoproteins and, therefore, the genes involved in pyoverdine biosynthesis were upregulated as it is required for bacterial survival in mammalian hosts (77–81). Cystic fibrosis mucus provides carbon and energy sources including mucins, DNA, free amino acids, glucose, lactate, and ions for the growth of *P. aeruginosa* during infection (86, 87). The pentose phosphate pathway is involved in the breakdown of glucose to derive energy for bacterial survival and provides intermediates for the biosynthesis of amino acids, nucleotides, and cell wall constituents, and, therefore, the genes involved in the utilization of these intermediates for biomolecule synthesis were found to be upregulated.

Module 28 was enriched in bacteriocin biosynthetic process, which was identified to be upregulated. Bacteriocins are antimicrobial peptides that are synthesized by *P. aeruginosa* to kill non-immune bacterial cells (88, 89) for self-preservation and competitive advantage. Increased biosynthesis of bacteriocin in cystic fibrosis lungs indicates bacterial strategy to overcome competition from other bacterial strains. We found upregulation of GO processes, including regulation of iron ion transport, siderophore transport, and secretion, in module 30. This module was also enriched in a two-component system, which was found to be upregulated in cystic fibrosis lungs. Two-component systems are mediators of signal transduction in diverse environments (90) and are required for bacterial virulence (91), so it is plausible that module 30 is critical for *P. aeruginosa* pathogenesis.

Module 31 was enriched in an upregulated process of siderophore uptake transmembrane transporter activity. Module 31 was also enriched in D-amino acid metabolic pathway. Module 36 was identified to be enriched in upregulated processes and pathways involved in anaerobic respiration, nitrate reductase activity, and nitrogen metabolism. A previous study has revealed that nitrate reductase activity is required for anaerobic growth of *P. aeruginosa* in cystic fibrosis lungs (92), and therefore, the upregulation of nitrate reductase activity and anaerobic respiration in module 36 indicates the possible role of this module in cystic fibrosis pathogenesis.

Modules 26 and 41 were enriched in bacterial type III secretion system that was upregulated, and module 42 was enriched in upregulated processes and pathways involved in siderophore pyochelin biosynthesis. Type III secretion system is used for host cell invasion and lysis (93, 94) and was also found to be upregulated in chronic wound infection, which indicates the importance of type III secretion system in bacterial pathogenesis. As modules 36 and 41 were also found to be enriched in DEGs in chronic wound infection, we posit that the differentially regulated processes and pathways represented by these modules are critical for different facets of *P. aeruginosa* pathogenesis. Iron, required for bacterial growth and survival, is not freely available in mammalian hosts, and therefore, bacteria such as *P. aeruginosa* synthesize siderophores including pyochelin and pyoverdine for chelating iron from host hemoproteins (77–81). As modules 23, 30, 31, and 42 were enriched in siderophores’ synthesis and transport, these modules could be playing an important role in the *P. aeruginosa* pathogenesis in cystic fibrosis lungs.

We further identified a module (#44) that is not enriched in DEGs but comprises over 65% genes that are either DEGs (50%) or LDEGs (16.66%) (Table 2; Web Material File 9). The LDEGs in this module are associated with a large fraction of genes that are DEGs, indicating their potential roles in pathogenesis. We performed GO and pathway enrichment analysis to understand the role played by this module in bacterial pathogenesis. This module was enriched in copper binding (GO process) but was not enriched in any pathways. Therefore, the role played by this module in *P. aeruginosa* pathogenesis could not be clearly discerned.

### Exposure of PAO1 to azithromycin

We mapped DEGs detected during the exposure of PAO1 to azithromycin onto the modules of the PAO1 network and identified 14 modules (3–5, 7, 8, 13, 15, 19, 26, 33, 38, 39, 41, 43) that were enriched in DEGs and contained a large number of (over 65%) DEGs or LDEGs (Table 3; Web Material File 9). To decipher the roles of these modules during the treatment of PAO1 with azithromycin, we performed GO and pathway enrichment analyses (Web Material File 10). GO and pathway enrichment analyses revealed upregulation of lipopolysaccharide biosynthesis, cell cycle (DNA replication, homologous recombination, and mismatch repair), and amino acid biosynthesis in module 3. Azithromycin inhibits bacterial protein synthesis (69). As proteins and enzymes are required for bacterial growth, multiplication, and metabolism, it is likely that module 3 aids bacterial growth and metabolism during the exposure of PAO1 to azithromycin. GO enrichment of downregulated processes such as bacterial chemotaxis and quorum sensing was observed in module 4. Upregulated processes identified to be enriched in this module include translation and ribosome. We found enrichment of pathways involved in biofilm formation, two-component system, and quorum sensing in module 4, which were downregulated. GO processes enriched in module 5 include those with downregulation in type 4 pilus biogenesis and assembly, pilus dependent motility, and chemotaxis. Pathways identified to be enriched and downregulated in module 5 include bacterial chemotaxis and two-component system. In module 7, GO processes found to be enriched include phenazine biosynthesis and alginate biosynthesis, both of which were downregulated. We also found enrichment of KEGG pathways related to quorum sensing and phenazine biosynthesis in module 7, which were both downregulated. As azithromycin dysregulates pili-mediated chemotaxis (95), two-component system (96), biofilm formation (97), phenazine biosynthesis (98), quorum sensing, and alginate biosynthesis (99, 100), enrichment of processes associated with these modules supports our model’s effectiveness in unraveling processes that are relevant during the treatment of PAO1 with azithromycin.

**TABLE 3:** DEG-enriched modules with over 65% of genes identified as either DEGs or LDEGs upon exposure of PAO1 to azithromycin

Module	DEG count	LDEG count	Genes (DEGs + LDEGs) (%)	Fold enrichment	Fisher’s test (*P*-value)	Enriched/non-enriched
3	252	3	0.652	1.158	2.518e-33	Enriched
4	284	0	0.825	1.484	6.146e-76	Enriched
5	110	11	0.668	1.092	2.370e-12	Enriched
8	61	0	0.802	1.443	7.986e-16	Enriched
13	48	1	0.710	1.250	6.593e-09	Enriched
15	58	0	0.865	1.556	3.520e-18	Enriched
19	35	6	0.694	1.066	1.733e-04	Enriched
26	19	0	0.760	1.366	5.592e-05	Enriched
33	15	1	0.800	1.348	4.975e-04	Enriched
38	9	2	0.785	1.156	4.494e-2	Enriched
39	12	1	0.928	1.541	1.489e-04	Enriched
41	11	0	0.846	1.521	3.658e-04	Enriched
43	9	0	0.692	1.244	1.659e-02	Enriched
7	69	13	0.656	0.992	4.622e-06	Non-enriched
44	6	4	0.833	0.899	0.364	Non-enriched

Cytoplasmic translation and ribosome were found to be upregulated and enriched GO and pathway terms, respectively, in module 8. GO processes found to be enriched in module 13 include transcriptional processes. We observed enrichment of translation and ribosome among the GO terms in module 15, with corresponding genes for both upregulated in this module. We also observed enrichment of pathway involved in biofilm (downregulated), as well as ribosomal pathways (upregulated) in this module. Elevated expression of ribosomal genes and genes involved in translation in the response to azithromycin treatment was also reported by another study (101) and could be indicative of bacterial mechanism to compensate for impaired translation during the azithromycin treatment. Module 19 was identified to be enriched in arginine biosynthesis pathway, which was downregulated. GO and pathway analysis revealed enrichment of type 3 bacterial secretion system (upregulated) in module 26. GO terms found to be enriched with the respective genes downregulated in module 33 include protein transport, xenobiotic transport, and efflux transmembrane transporter activity. We further identified enrichment of quorum sensing pathway with corresponding genes downregulated. GO process identified to be enriched in module 38 involves oxidoreductase activity that was downregulated. In module 39, we observed GO and pathway enrichment of type VI bacterial secretion system and biofilm formation, respectively, which were downregulated during the exposure to azithromycin. We observed enrichment of a process involved in bacterial type 3 secretion system (for both GO and pathway) in module 41, which was upregulated during the treatment. Contrary to the findings of previous studies (95, 102), we found upregulation of genes that are components of type III secretion system as also reported by a recent study (101). Azithromycin reduces quorum sensing in *P. aeruginosa* (100). Studies have revealed that the downregulation of quorum sensing (identified in modules 4 and 33) in turn regulates bacterial virulence including type VI secretion system (103, 104). As module 39 was observed to be enriched in type VI secretion system, with corresponding genes downregulated, it is plausible that the functionalities of this module are dysregulated during the azithromycin treatment. GO processes enriched in module 43 include phenazine and secondary metabolite biosynthesis, both of which were downregulated. We also observed the enrichment of quorum sensing pathway (downregulated) in this module. As azithromycin attenuates phenazine biosynthesis (22) and quorum sensing (100), module 43, representing such processes or pathways, is likely to be impacted during the treatment of PAO1 with azithromycin.

**Fig 5:**
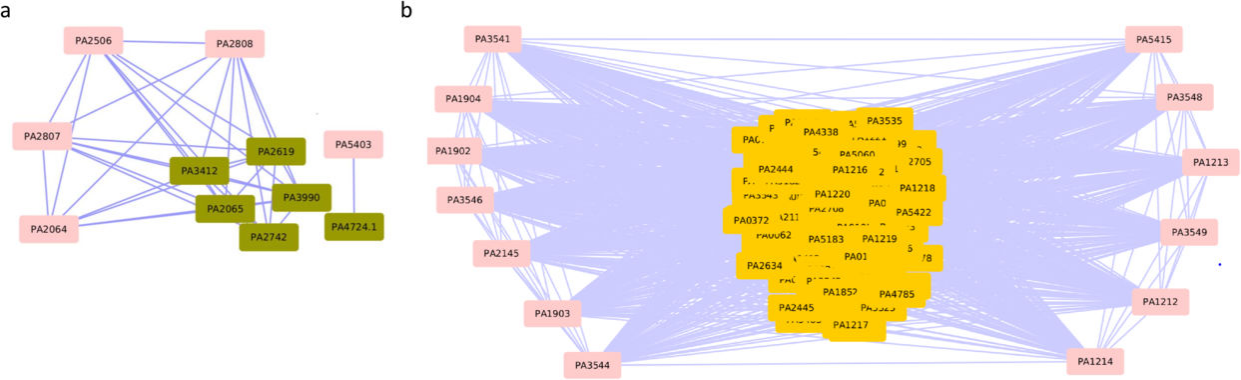
(**a**) Module 44 and (b) module 7 are not significantly enriched in DEGs but comprise over 65% of genes that are either DEGs or LDEGs in PAO1 during its treatment with azithromycin. LDEGs in each module are highlighted in pink.

Interestingly, we found two modules, labeled 7 and 44 (Fig. 5a and b), which are not enriched in DEGs but comprise over 65% of genes that are either DEGs (55% and 50%, respectively) or LDEGs (10% and 33.33%, respectively) (Table 3; Web Material File 9). The association of these LDEGs with a relatively larger fraction of DEGs in these modules indicates their potential roles in the response to azithromycin. We performed GO and pathway enrichment analysis to decipher the plausible role of this module in response to azithromycin treatment. We identified GO and pathway enrichment of quorum sensing and metabolic pathways in module 7. We observed GO enrichment of copper binding in module 44. No pathways were found to be enriched in this module, and thus the role played by this module during the azithromycin treatment of PAO1 is not yet clear.

Considering that modules 7 and 44 are not enriched in DEGs associated with the exposure to azithromycin but comprise ~65% and ~83% genes, respectively, that are DEGs or LDEGs, these modules will likely be ignored by standard protocols but are prioritized by our systems-level approach presented here and, therefore, are potential candidates for further investigation to establish their roles in the PAO1 response to azithromycin, either in the regulation of bacterial survival or in susceptibility during azithromycin treatment.

### Pathway annotations for LDEGs

#### Acute burn and chronic surgical wound infections

We searched for LDEGs in any known pathways using KEGG or the literature (Web Material File 11). In acute burn wound infection, no LDEGs were found in module 42. In chronic surgical wound infections, we found several upregulated LDEGs in module 8 including *PA0038, PA2883, PA3928*, and a downregulated gene *PA3752*, which are not associated with any known pathways. We identified upregulated genes, such as *PA0567,* which functions in cell envelope homeostasis (105), *PA1592,* which is within pathway implicated in biofilm formation and long-term infection (106), and *PA4738* and *PA5482,* which are involved in protection against osmotic stress (107). As pathogens encounter various stress factors during infection including osmotic stress that can interfere with cell envelope homeostasis (108, 109), the upregulation of the LDEGs known to function in envelope homeostasis and protection against osmotic stress is quite plausible. LDEG *cntM* encodes a protein involved in the biosynthesis of metallophore pseudopaline that plays a role in zinc and nickel uptake (110); this LDEG, present in module 31, was found to be upregulated during the infection. LDEG *PA4838*, upregulated during the infection, encodes regulation of zinc acquisition (111). Reactive oxygen species released by host during an infection participates in immune and inflammatory response against the infection and has harmful effects on the invading pathogen (112). As zinc ions are involved in bacterial DNA repair, response against oxidative stress (113–115), and biofilm formation (116), it is likely that genes involved in zinc uptake were activated in the chronic wound infection. Several LDEGs with elevated expression in module 42, such as *pchB*, *pchC*, *pchD*, *pchE*, *pchG*, and *PA4220*, were found to lie within pathways for pyochelin biosynthesis and metabolism (117). Mammalian hosts contain hemoproteins and extracellular proteins such as transferrin, lactoferrin, and ferritin (118–120), which bind iron, making it less freely available for bacterial pathogens. PAO1 produces siderophores such as pyochelin to chelate iron. As iron is required for the pathogen’s growth and survival (77–79) in a mammalian host, it is highly likely that pyochelin biosynthesis genes (*pch*) were upregulated during the PAO1 pathogenesis. LDEG *PA4222*, also found in the same module, plays a role in *P. aeruginosa* defense mechanisms. Several LDEGs, such as *PA5537*, encode proteins of yet unknown function.

#### Cystic fibrosis lung infection

We investigated the KEGG pathway database and literature for pathway annotations of LDEGs identified in the *P. aeruginosa* infection of cystic fibrosis lungs (Web Material File 11). LDEGs in module 9 are known to be involved in denitrification pathway (121, 122), which includes nitrous oxide reductase genes (*nosD* and *nosF*) and nitrite reductase genes (*nirC* and *nirH*). Expression of nitrous oxide reductase (*nos*) genes increased, while that of nitrite reductase (*nir*) genes decreased. As transcriptional profiling provides a snapshot of the pathways or parts of pathways affected at the time of collection of biological samples from a certain condition, it is plausible that some gene(s) of a pathway can get activated as the substrates required for the gene products are made available through the upstream genes, resulting in the synthesis of the downstream intermediates. When the expression of the upstream genes is no longer required to generate more substrates, their expression can decrease. Therefore, suppression of the expression of upstream *nir* genes and activation of downstream *nos* genes indicate the availability of abundant nitrous oxide that needs to be converted to dinitrogen via the denitrification pathway at that time point. As module 9 was found to be enriched in DEGs involved in denitrification, it is likely that this module plays a role in denitrification during the cystic fibrosis lung infection. We identified elevated expression levels of a gene (*PA0202*) involved in amino acid metabolism and two genes (*mdcC*, *acoB*) that function in carbon and energy metabolism (123) in module 14. As cystic fibrosis mucus provides carbon and energy sources (86, 87), which are utilized for bacterial growth, it seems plausible that these LDEGs are activated in cystic fibrosis lungs. We further identified three genes, *PA2422*, *PA4149,* and *PA4929*, which encode proteins with yet unknown function but could be involved in the *P. aeruginosa* pathogenesis. Module 23 harbored three LDEGs that displayed increased expression*—phzC2* that functions in phenazine biosynthesis, and *pvdF* and *fpvA* that are involved in siderophore (pyoverdine) biosynthesis (124–126). Phenazine is an important virulence factor required for biofilm formation in chronic infections (127). Previous studies have associated pyoverdine with the solubilization of iron from inorganic sources, chelation of iron from host iron-sequestering factors (128), and regulation of toxin secretion (129, 130) during infections, and therefore it is highly likely that these LDEGs were upregulated, as inferred from our network analysis, during the cystic fibrosis lung infection. In module 28, we observed increased expression of *pys2*, which is known to be involved in the killing of non-immune bacterial strains via the fpvA type I ferripyoverdine receptor (131). This gene was also found to be activated in module 23. Bacteriocin encoded by this LDEG might be required to overcome competition from other bacterial strains, supporting potential upregulation of this gene, as borne out by our analysis, during the colonization of *P. aeruginosa* in the lungs of cystic fibrosis patients. We further identified elevated expression of two other genes in module 28 (*PA1152* and *PA3741*), which encode uncharacterized proteins whose functions are not yet known. Increased expression levels of LDEGs involved in anaerobic respiration (*narK1*) and molybdopterin biosynthesis (*moaB1*) were observed in module 36. A previous study has revealed that biosynthesis of the molybdopterin cofactor is essential for nitrate reductase activity (132), which in turn facilitates denitrification (anaerobic respiration) required for the survival of *P. aeruginosa* in the microaerobic environment of cystic fibrosis lungs. *pscP* involved in bacterial type III secretion was harbored by module 41 and inferred as LDEG with upregulation. Type III secretion system is known to be involved in the invasion and lysis of host cells (93, 94).

#### Exposure of PAO1 to azithromycin

Similarly, we examined known pathways using KEGG or the literature to ascertain the presence of LDEGs identified during the exposure of PAO1 to azithromycin in any known pathways (Web Material File 11). LDEGs *nuoH, nuoI,* and *nuoJ* genes in module 3, which displayed elevated expression during the azithromycin exposure, lie within a pathway involved in anaerobic respiration. Previous studies have not revealed any association between anaerobic respiration and azithromycin; therefore, the role of these activated genes in their response to the azithromycin treatment is not immediately clear. LDEGs *hxcW* and *hxcT* in module 5 had elevated expression and encode a bacterial secretion system; on the other hand, *hxcU* and *hxcP* that encode type II secretion system were downregulated in this module. *lapA* gene, which encodes a biofilm matrix protein, was found to be downregulated upon exposure to azithromycin. LapA is secreted via the *hxc* genes (133). As *hxc* genes are involved in the secretion of LapA, which plays a role in PAO1 biofilm formation, it is likely that the expression of these genes was affected by azithromycin, which inhibits biofilm formation (97). Other downregulated LDEGs include *agt2* involved in glycolipid biosynthesis during phosphorus stress (134), *PA2804* known to be activated during phosphate limitation or starvation (135), *eddA* involved in extracellular DNA degradation that leads to biofilm dispersion (136), and *PA1873*, which is not associated with any known pathway. As azithromycin interferes with the outer membrane (137) and is also known to inhibit PAO1 biofilm formation (97), it is likely that *agt2* and *eddA* genes involved in membrane integrity maintenance and biofilm dispersion, respectively, were suppressed in expression during the azithromycin treatment, as revealed by our network analysis.

LDEGs found to be upregulated in module 13 include *PA4596* that encodes esrC, an envelope stress-regulated repressor of the *mexCD-oprJ* multidrug efflux operon (138). As azithromycin interacts with the outer membrane and leads to the displacement of Mg^2+^ and Ca^2+^ ions existing between bacterial lipopolysaccharide layers (137), *esrC* gene is likely to be activated and results in the suppression of *mexCD-oprJ* multidrug efflux pump operon involved in azithromycin efflux and resistance against the macrolide (139). LDEG *PA0141* in module 19 encodes a hypothetical protein involved in RNA degradation, and in the same module, LDEG *PA4577* encodes a hypothetical protein that is not associated with any known pathway; both displayed elevated expression. We identified a downregulated LDEG in module 33, *PA3237*, which is regulated by hydroxyl radicals (140). LDEGs *PA1896* and *PA1897* of module 38 were found downregulated and upregulated, respectively. These genes are part of an operon regulated by QscR, which leads to dampening of quorum sensing signals (141). QscR is a regulator of quorum sensing that delays the activation of quorum sensing genes by upregulating *PA1895-PA1897* operon (141). *qscR* was one of the upregulated DEGs during azithromycin treatment of PAO1. Activation of *qscR* could lead to upregulation of PA1897, which in turn dampens quorum sensing signals. Although PA1897 was found to be upregulated, PA1896 was identified to be downregulated, which could potentially indicate dysregulation of this quorum sensing operon by azithromycin. In module 39, we identified an LDEG, *hcpA*, that functions in biofilm formation and bacterial secretion system and displayed lower expression relative to the control. As azithromycin attenuates PAO1 biofilm formation (97), the expression of *hcpA* could potentially be suppressed upon exposure to azithromycin.

#### Use of Tn-seq data to identify LDEGs contributing to the fitness of PAO1

To decipher the role of LDEGs in imparting fitness to PAO1, we examined the transposon sequencing (Tn-seq) data from a murine wound infection study (69). We found LDEGs involved in pyochelin biosynthesis (*pchB, pchD, pchE,* and *pchG*) and type III secretion system (*pscO, pscQ*) among the fitness contributing genes deciphered by Tn-seq during the chronic wound infection (Table 4). Pyochelin is known to chelate iron from mammalian hemoproteins and contributes to bacterial fitness in mammalian hosts (77–81). Type III secretion system is used by *P. aeruginosa* to inject toxin proteins including ExoS into host cells and is thus required for efficient pathogenesis of the host. As pyochelin biosynthesis and type III secretion system are important virulence factors required for efficient pathogenesis, DEGs identified by our approach help in deciphering molecular players that may otherwise get overlooked by traditional approaches for differential gene expression analysis.

**TABLE 4:** LDEGs contributing to the fitness of PAO1 in *in vivo* chronic wound infections

Module	Gene name	Locus tag	Expression fold change	Expression *P*-value	Mutant abundance fold change	Mutant abundance *P*-value
41	*pscQ*	*PA1694*	9.9	0.046	9.23	2.5e-03
41	*pscO*	*PA1696*	13.47	0.031	13.40	2.0e-03
42	*pchB*	*PA4230*	15.07	0.11	17.00	5.0e-03
42	*pchD*	*PA4228*	47.6	0.14	4.21	8.5e-03
42	*pchE*	*PA4226*	36.82	0.025	3.74	2.2e-02
42	*pchG*	*PA4224*	22.29	0.34	5.86	6.5e-03

#### Use of machine learning-based perturbome data to identify LDEGs associated with several stress conditions in PAO1

We revisited PAO1 genes that were reported to be associated with several stress conditions including antibiotics, drugs, light, osmotic stress, oxidative stress, and temperature (heat and cold) by a previous study (67) based on a machine-learning approach (67). We examined the overlap between these genes and those found by our approach during the treatment of PAO1 with azithromycin. We found that two LDEGs (*nuoI* and *nuoJ*) identified by our pipeline (Web Material File 11) were listed as genes involved in several stress-related conditions by this study (67). *nuoI* and *nuoJ* displayed elevated expression during azithromycin treatment (Web Material File 11) and are known to be involved in anaerobic respiration pathway.

To summarize, the current protocols for differential gene expression analysis use a conservative approach based on expression fold change and statistical significance test (3–6). Fold change does not take variability into account and does not guarantee reproducibility of the results for differential gene expression analysis (1, 2). These protocols tend to generate numerous false negatives. Therefore, in this study, we have attempted to circumvent this false negative problem by integrating the traditional approach for differential gene expression analysis with a gene co-expression network-based approach. We demonstrated the usefulness of this novel pipeline using *P. aeruginosa* PAO1 transcriptomic data from previous studies (63, 64, 70). As *P. aeruginosa* is a well-known multidrug resistant pathogen that is implicated in several diseases including urinary, lung, and wound infections, we applied our pipeline to decipher genes and pathways differentially regulated during pathogenesis and exposure to antibiotics. We obtained DEGs by performing differential gene expression analysis of PAO1 genes in a cystic fibrosis lung environment, as well as from previous studies (63, 64). We then mapped DEGs obtained from an RNA-Seq experiment onto the PAO1 gene co-expression network. This enabled identification of modules enriched in DEGs and also inference of LDEGs and the pathways they lie in, both known and unknown, during pathogenesis and antibiotic treatment. We identified LDEGs based on their association with DEGs in DEG-enriched modules that harbored a large majority (over 65%) of genes that were either DEGs or LDEGs. This guilt-by-association approach (35–37) has been widely used by researchers to infer gene functions and pathways in different organisms including fungi, humans, and plants (10, 11, 24–34). We exploited this further in a novel systems-level framework to identify yet unknown genes, pathways, and networks of genes involved in PAO1 pathogenesis and in the response of PAO1 to azithromycin treatment (59, 61).

Using gene co-expression network for functional inferences has its own set of biological as well as computational limitations. Genes that have similar expression patterns may not necessarily have similar or related functions (142), and genes that have related functions may not have similar expression patterns due to different levels of post-transcriptional and other regulations (143). Different thresholds were used by the Dynamic Tree Cut algorithm in WGCNA to generate modules (144). Different parameters of the Dynamic Tree Cut algorithm including height and shape parameters affect gene co-expression network construction, network connectivity, and the number of modules in the network (144, 145). WGCNA further requires users to choose a soft-thresholding power to construct a network that may approximate a scale-free topology. As we used different parameter thresholds for the construction of this network and subsequently detection of modules, the network may be biased by the selection of these thresholds. As there is no known protocol to optimally select the WGCNA parameters and thresholds that detect modules with high biological conformity, different approaches have been used to validate the robustness of the network. Some studies have used prior knowledge from databases for the construction of robust gene co-expression networks (12, 146–148). Others have used a large number of samples to construct co-expression networks containing high-confidence gene interactions (26, 149). We have constructed the PAO1 gene co-expression network using RNA-Seq data sets for PAO1 grown in diverse conditions, similar to studies (16, 19, 26) that have used expression profiles from several conditions for the construction of co-expression networks. Enrichment of operon genes in the modules placed further confidence over the network (Web Material File 3).

### Concluding remarks

Standard differential gene expression analysis pipelines that use expression fold change and statistical significance test are often invoked to investigate genes that express differentially during pathogenesis in bacterial pathogens. Our study highlights the importance of the systems-level approach, such as a gene network, to decipher biologically significant entities (e.g., differentially regulated genes or modules) that could fall below the detection threshold established in the standard protocols and could thus be used in concert with a conventional differential gene expression analysis pipeline to comprehensively identify genes or pathways differentially expressed during pathogenesis in bacterial pathogens.

In application to *P. aeruginosa* expression data obtained from acute and chronic wound infections as well as from infections in cystic fibrosis lungs, our network-based pipeline identified several unreported genes that are likely differentially expressing and function in yet unknown pathways. Many of these genes could potentially be involved in novel pathological pathways that have not yet been identified. Our analysis also aided in the identification of genes that play roles in known pathways but their functions in pathogenesis remain to be elucidated. Similarly, our approach, when applied to the PAO1 expression data obtained from the exposure of PAO1 to azithromycin, identified several previously unreported genes that have not yet been associated with any known pathways. Of course, as with any statistical approaches, these novel findings have to be verified using experimental assays. Future studies could, therefore, focus on validating novel genes or pathways identified to be potentially involved in the PAO1 pathogenesis and response to azithromycin.

## Data Availability

The associated data sets are available at the project’s GitHub website: https://github.com/Ronika19/PAO1_Network. All other data sets are provided with the article and supplemental materials. The NCBI accession numbers of *P. aeruginosa* PAO1 RNA-Seq data sets that were used for discovering novel genes differentially expressing during pathogenesis and response to azithromycin are SRP033652 and SRP264926, respectively.
